# Does body posture influence hand preference in an ancestral primate model?

**DOI:** 10.1186/1471-2148-11-52

**Published:** 2011-02-28

**Authors:** Marina Scheumann, Marine Joly-Radko, Lisette Leliveld, Elke Zimmermann

**Affiliations:** 1 Institute of Zoology, University of Veterinary Medicine Hannover, Bünteweg 17, D-30559 Hannover, Germany; 2 Center for Systems Neuroscience, Bünteweg 17, D-30559 Hannover, Germany

## Abstract

**Background:**

The origin of human handedness and its evolution in primates is presently under debate. Current hypotheses suggest that body posture (postural origin hypothesis and bipedalism hypothesis) have an important impact on the evolution of handedness in primates. To gain insight into the origin of manual lateralization in primates, we studied gray mouse lemurs, suggested to represent the most ancestral primate condition. First, we investigated hand preference in a simple food grasping task to explore the importance of hand usage in a natural foraging situation. Second, we explored the influence of body posture by applying a forced food grasping task with varying postural demands (sit, biped, cling, triped).

**Results:**

The tested mouse lemur population did not prefer to use their hands alone to grasp for food items. Instead, they preferred to pick them up using a mouth-hand combination or the mouth alone. If mouth usage was inhibited, they showed an individual but no population level handedness for all four postural forced food grasping tasks. Additionally, we found no influence of body posture on hand preference in gray mouse lemurs.

**Conclusion:**

Our results do not support the current theories of primate handedness. Rather, they propose that ecological adaptation indicated by postural habit and body size of a given species has an important impact on hand preference in primates. Our findings suggest that small-bodied, quadrupedal primates, adapted to the fine branch niche of dense forests, prefer mouth retrieval of food and are less manually lateralized than large-bodied species which consume food in a more upright, and less stable body posture.

## Background

In humans it is believed that handedness is related to brain lateralization of language and other cognitive functions. Therefore, handedness has become a major interest in evolutionary research. Approximately 90% of the human population are right-handed independent of culture [1,2]. Fossil records and recent findings in great apes indicate that right-handedness evolved early in human evolution [2,3]. However, to date, the evolution of primate handedness, and thus, the origin of human handedness, is still unclear. Recent studies of handedness in primates revealed that hand preference is influenced by a number of different factors including body posture, sex, age, task difficulty, task complexity and experience [4,5], making it difficult to reconstruct its evolution.

To date, there are two major hypotheses related to the influence of body posture. First, the *postural origin hypothesis *by MacNeilage et al. [6] proposes that primate handedness patterns evolved with structural and functional adaptations for feeding. As a first step in primate evolution, left-hand preference evolved for visually guided reaching (unimanual predation), whereas the right hand was used for postural support. This holds especially true for arboreal prosimians and is supported by the fact that most prosimian species exhibit a left-hand preference at population level [7]. As a second step, with evolution of a more terrestrial life style, the right hand was no longer necessary for postural support and became specialized for object manipulation and bimanual coordination in higher primates. Second, the *bipedalism theory *proposes that the shift from a stable quadrupedal to an unstable bipedal posture necessitated higher balance control which is reflected in an increased cerebral lateralization [8,9]. Thus, primates should show a higher degree of manual lateralization in a bipedal position than in a quadrupedal one.

In this study we will test the two hypotheses by investigating the effect of different body postures on hand preference in an ancestral primate model while controlling for the level of difficulty. Several experimental studies have investigated the effect of posture on hand preference during reaching in other non-human primates; food items were placed at different heights relative to the cage floor to obtain specific body postures. However, most of these studies focused on the comparison of quadrupedal versus bipedal postures (e.g. [9-16]).

Ape studies showed a shift to a greater use of the right hand in bipedal versus quadrupedal reaching for chimpanzees, orang-utans and gorillas [13,14], whereas the results from bonobos are contradictory. Hopkins and colleagues [13] found that bonobos also showed a stronger right-hand preference in bipedal than in quadrupedal postures, whereas Vleeschouwer and colleagues [11] found an increase in left-hand preference when the animals shifted from a seated to a bipedal via a quadrupedal posture. In a recent study Braccini and collegues [15] used a unimanual tool-use task (subject had to remove peanut butter out of a tube with a stick) to test hand preference in three experimentally induced postures (seated, bipedal supported and unsupported). The strength of hand preference increased from seated to bipedal posture but the direction of hand preference was not affected. In gibbons left-handedness has been found in bipedal tasks, whereas no population level handedness has been found for quadrupedal tasks [14].

In Old World monkeys a shift towards right-handedness with increasing upright body posture was reported for rhesus macaques (bipedal versus quadrupedal: [9]) and Gray-cheeked mangabies (sat versus biped and clung:[17]). Further, Campbell's monkeys showed significant differences in the strength, but not the direction, of hand preference between different postural tasks [18]. The strength was weaker for the triped (= quadrupedal) task than for the biped, clung and sat tasks.

In New World monkeys a shift to right-hand preference in bipedal versus quadrupedal reaching tasks has been noted for tufted capuchins [12,19,20]. In contrast, in squirrel monkeys, King & Landau [16] observed a trend to left-handedness for bipedal versus quadrupedal reaching and a trend to right-handedness for vertical clinging. For Callitrichinae no influence of body posture on the direction of hand preference has been observed [21-23]. An increase of the strength of laterality from stable horizontal to unstable bipedal or clinging posture has been reported for tufted capuchins [12], squirrel monkeys [21,24] cotton-top tamarins [21] and common marmosets [23,25,26].

For prosimians, a shift to left-hand preference and an increase in the strength of hand preference for bimanual versus quadrupedal tasks has been observed in Senegal bushbabies [8,27]. Additionally, ruffed lemurs show a shift to left hand preference for tasks of extreme postural adjustment versus free foraging tasks [28]. In contrast, no effects of postural adjustment on the strength and direction of hand preference were found in Garnett's bush babies [29], South African lesser bushbabies and gray mouse lemurs [10].

All in all, primates show a tendency towards increasing the strength of hand preference from a stable reaching position (quadrupedal, sit) to an unstable reaching position (bipedal, cling). Results for the direction of hand preference are not so clear but indicate an evolutionary trend from left-handedness in prosimians to right-handedness in great apes.

In this study we investigated hand preference of an ancestral primate model, the gray mouse lemur [30]. The mouse lemur is a suitable model for evolutionary research because its phylogenetic position in Primates provides the first insight into the evolutionary roots of primate manual lateralization. In addition, its lissencephalic brain organization compared to anthropoid primates, makes it a useful model for neurobiological research [31]. The gray mouse lemur is a small-bodied, quadrupedal, arboreal, nocturnal primate species living in the fine branch niche of the Malagasy forests [30]. First, we investigated hand preference in a simple food grasping task (SGT), representing the natural foraging environment, to estimate the importance of hand usage during natural foraging based on a large sample size. In a previous study it was shown that gray mouse lemurs seem to prefer to use their mouths to pick up raisins, but this was based on a small sample size (N = 8; [32,33]). Second, we compared hand preference for the first time in prosimians using four forced food grasping tasks (FGT) which varied in their postural demands/postural stability (FGT-sit, FGT-biped, FGT-cling, FGT-triped). In the FGT tasks a subject has to retrieve mealworms out of a box reaching one hand through a grid which prevents usage of the mouth. To date, handedness data for mouse lemurs have only been available for a seated posture [10,34,35] and for a small sample size for the biped posture (N = 8, [10]) indicating individual but no population level handedness. Therefore, we tested hand preference for the first time for the cling and triped postures and for a large sample size for the biped posture. Third, we investigated whether the different postural demands vary in their level of difficulty and whether this fact affects the hand preference of gray mouse lemurs. Since the assumed difficulty of the task itself, as perceived by the human experimenter, may not match the difficulty experienced by the species tested [36], we used the percentage of successful hand grasps (= success rate) as an objective measurement of the level of difficulty.

All in all, we investigated the following three questions: First, do mouse lemurs prefer to use their hands to catch mealworms in a natural foraging situation? Second, does body posture have an influence on the direction and strength of hand preference? Third, does the level of difficulty of the postural demand tasks have an influence on the direction and strength of hand preference?

## Methods

### Subjects

We tested 56 gray mouse lemurs (*Microcebus murinus*, 24 males, 32 females) of our breeding colony, housed in the animal facility of the Institute of Zoology, University of Veterinary Medicine Hannover (for details on housing conditions see [37]). All subjects had been born in captivity. Their ages ranged from 7 months to 9 years. The experiments were licensed by the Bezirksregierung Hannover, Germany (reference number: 509c-42502-03/660) and complied with the Animal Care guidelines and the applicable national law.

### Experimental set-up

**Figure 1:**
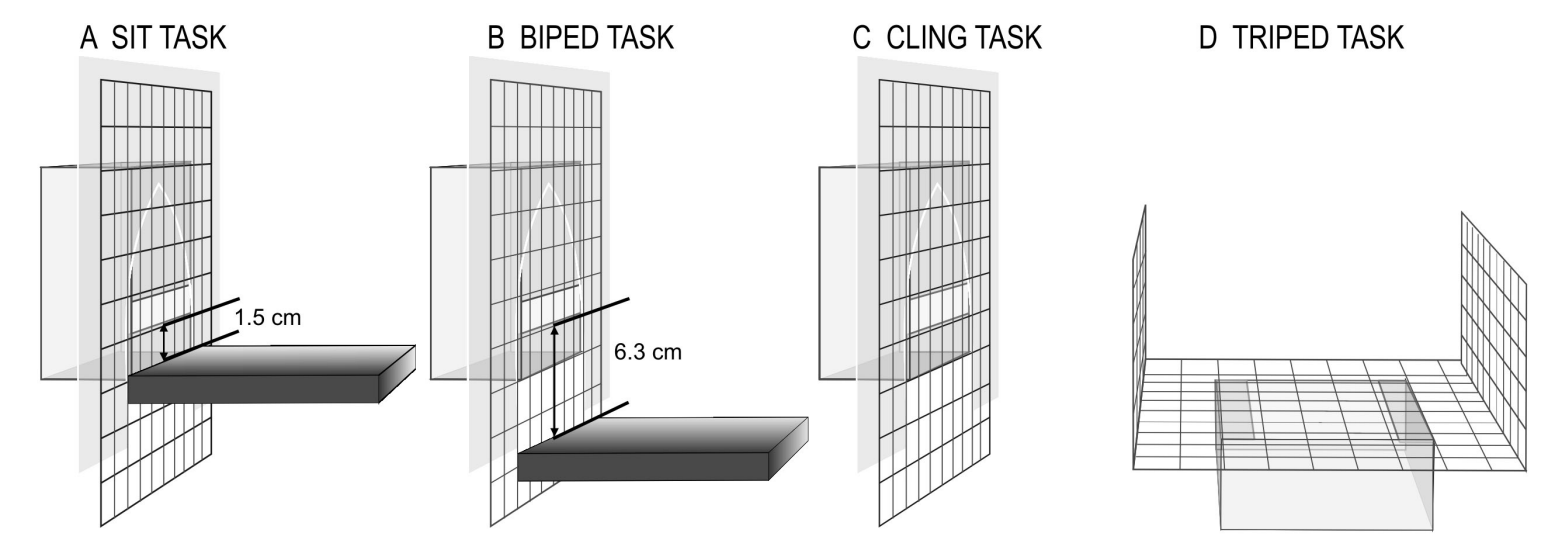
**Experimental set-up for the four postural tasks (FGT-sit, FGT-biped, FGT-cling and FGT-triped)**. A plastic shield was used to standardize the position of the subject in front of the transparent box for the FGT-sit, FGT- biped and FGT-cling task.

Each mouse lemur was tested alone in a test cage [Ebecco stainless steel cage for marmosets, 80 cm × 87 cm × 50 cm] in a separate testing room. The cage was equipped with two wooden bars and a nest box. For the simple food grasping task (SGT) a food bowl (diameter: 10 cm) was placed into the test cage. For the forced food grasping tasks (FGT) either a transparent box with a small opening (1x4 cm) was attached to the outside of the cage (FGT-sit, FGT-biped, FGT-cling) or a plastic box was placed below the grid ground (FGT-triped; Figure 1). This prevented the animals from using their mouth so that they were forced to grab with one hand through the small openings between the bars. The subjects' behavior was videotaped using a digital camcorder [Sony DR-TRV 22E PAL or SONY Camcorder DCR-SR75E, Nightshot]. The camera was connected to a monitor outside the testing room where the experimenter sat and observed the subjects.

### General Procedure

Each session was conducted at the beginning of the activity period for each subject.

For each session a subject was removed from its home cage, placed in a new nest box attached to the test cage in the testing room. For each session 10 mobile (SGT) or immobile mealworms (FGT) were placed in the food bowl (SGT) or plastic box (FGT). Each subject was tested for 15 minutes or until the subject had eaten all food items. A session started as soon as the door to the testing room had been closed to rule out any influence of the experimenter. An experimental task consisted of three sessions on three separate days. Thus, a subject needed a minimum of three days (= three sessions) to complete one experimental task. In cases where the subject retrieved less than 9 mealworms per session a fourth session was conducted to increase the number of grasping events.

### Experimental tasks

#### Simple food grasping task (SGT)

In the SGT task, we collected data for familiar actions belonging to the natural repertoire of the subjects. For each session we scattered 10 living mealworms on the bottom of a food bowl and the subjects were allowed to pick up the food items either with their hands or with their mouth or with a combination of both. This task was performed by 37 gray mouse lemurs (15 males, 22 females; see Additional file 1: Movie SGT).

#### Forced food grasping tasks with variation in postural demands (FGT)

To test for the effect of postural demands we conducted four forced food grasping tasks: FGT-sit, FGT-biped, FGT-cling, FGT-triped. In the FGT a subject had to use one of its hands to grab immobile mealworms (mealworms had to be immobilized to prevent them from crawling out of the transparent box) through a grid (grid size: 1 × 1 cm) and a small opening (1 × 4 cm) in a transparent box (FGT-sit, FGT-biped, FGT-cling), or through a grid into a plastic box below the ground (FGT-triped; Figure 1; see Additional files 2, 3, 4 and 5: Movie FGT-sit, Movie FGT-biped, Movie FGT-cling, Movie FGT-triped). The grid prevented the animals from using their mouth, forcing them to grab with one hand inside it. To induce different postural demands the transparent box was fixed at different heights to the wooden bar/floor (Figure 1).

For the FGT-sit task the opening of the transparent box was fixed at a distance of 1.5 cm from the wooden bar. The subject could sit on its hind legs while manipulating the food items with both hands. This task was performed by 54 gray mouse lemurs (23 males, 31 females; see Additional file 2: Movie FGT-sit). For this task we included data from 44 subjects already published by Scheumann and Zimmermann [34] and Leliveld et al. [35] to increase the sample size. From these data we included the first three sessions to keep the number of sessions comparable throughout the study. The 10 new subjects were born or available after the previous two studies had finished.

For the FGT-biped task the opening of the transparent box was fixed at a distance of 6.3 cm from the wooden bar. The subject had to stand on its hind legs and stretch its body while manipulating the food items with both hands. This task was performed by 31 gray mouse lemurs (13 males, 18 females; see Additional file 3: Movie FGT-biped).

For the FGT-cling task the opening of the transparent box was fixed on the grid of the cage. The transparent box was positioned in such a way to prevent the subject from coming into contact with the ground while taking the food items. The subject had to cling onto the grid while manipulating the food items. This task was performed by 31 gray mouse lemurs (13 males, 18 females; see Additional file 4: Movie FGT-cling).

For the FGT-triped task, a plastic box was fixed below the grid. Thus, when the subject picked up a food item, both feet and one hand touched the ground while the other hand grasped the mealworm. This task was performed by 29 gray mouse lemurs (12 males, 17 males; see Additional file 5: Movie FGT-triped).

For task comparison 27 gray mouse lemurs (11 males, 16 females) were used which performed all four postural tasks.

### Data and video analysis

When the experimental tasks had been videotaped using the Sony DR-TRV 22E PAL, we digitized all videotapes using InterVideo WinDVD creator 2. When experimental tasks had been recorded using Sony DCR-SR75E, the existing digital files were transferred to an external hard disk. We conducted a frame-by-frame analysis (25 frames/second) in Interact 3.1. (Mangold International GmbH).

For the SGT task, we recorded whether the subject used its mouth alone, its hand alone or a combination of both. Mouth alone was defined as occurring when the subject picked up the mealworm without using its hands. The hands were either on the edge of the bowl or on the bottom with no contact to the food item. Hand alone was defined as occurring when the subject picked up the mealworm without using its mouth. That means the subjects transferred the food item to the mouth after the item was no longer in contact with the ground. A combination of hand and mouth was coded if the two other behaviors were excluded, meaning subjects made a whole body movement and lunged at the food item with mouth and hands simultaneously. For the FGT tasks, we recorded the hand (right or left) the subject used to retrieve mealworms from the transparent box.

To measure the hand spontaneously chosen for a specific task (= hand preference), we analyzed the first grasp of each grasping bout. A grasping bout started with the first grasp of the subject and ended when it successfully retrieved a mealworm. A hand was considered to be successful when it had picked up one or more mealworms out of the box. A maximum of 10 grasping bouts (= 10 mealworms) could be analyzed per session. If the mouse lemur retrieved one or more mealworms out of the box successfully, it ate them before starting a new grasp. Therefore, the first grasps of each grasping bouts can be considered as independent from each other.

### Statistical analysis

We calculated the handedness index (HI) for each subject according to the formula HI = (number right - number left)/(number right + number left) [38]. The outcome of this formula can range from -1 to 1, with positive values reflecting right-hand bias and negative values reflecting left-hand bias. We additionally used the absolute HI (ABS-HI) value of each subject to compare the strength of the lateralization irrespective of direction.

We tested whether subjects used one hand more often than expected by chance using the Binominal test with 50% chance level. We defined animals as left- or right-handers or ambiguous: right-handers - subjects used the right hand significantly more often than expected by chance (positive handedness index), left-handers - subjects used the left hand significantly more often than expected by chance (negative handedness index), ambiguous - subjects did not use one hand significantly more often than expected by chance.

According to a Kolmogorov-Smirnov test, our data differed significantly from a normal distribution. For this reason, we used nonparametric tests (two-tailed). To explore whether a significant majority of the population was lateralized, we used a Chi-Square test with the number of left, right, and ambiguously handed individuals to test if this distribution differed significantly from chance (25:25:50, [39]). To test if the population showed a lateralization towards the right or the left hand, a Binomial test was conducted to test whether significantly more subjects used the right hand than expected by chance (50:50). Additionally, we performed a one-sample t-test on the HI score to investigate handedness at population level as is commonly done in the literature [4].

To explore sex differences we compared the HI and ABS-HI of males and females using the Mann-Whitney-U test. To explore age effects we correlated the HI and ABS-HI with the age of the subjects using a Spearman correlation.

To investigate the effect of postural demands we compared the HI and ABS-HI between the four postural tasks using the Friedman test. Further, we compared the number of lateralized subjects between the four postural tasks using the Cochran's Q test. We used the Spearman correlation to examine the relationship between the HI and ABS-HI for the four postural tasks.

To evaluate the level of difficulty of the postural demand tasks we calculated the percentage of successful hand grasps by dividing the number of successful hand grasps by the total number of hand grasps (= success rate). A success rate of 100% means that the subject was successful in all grasps. A success rate of 50% means that the subject successfully retrieved a mealworm in only half of all grasps.

All statistical tests were calculated using SPSS 17. We considered a result significant if p ≤ 0.05.

## Results

### Simple food grasping task (SGT)

**Figure 2:**
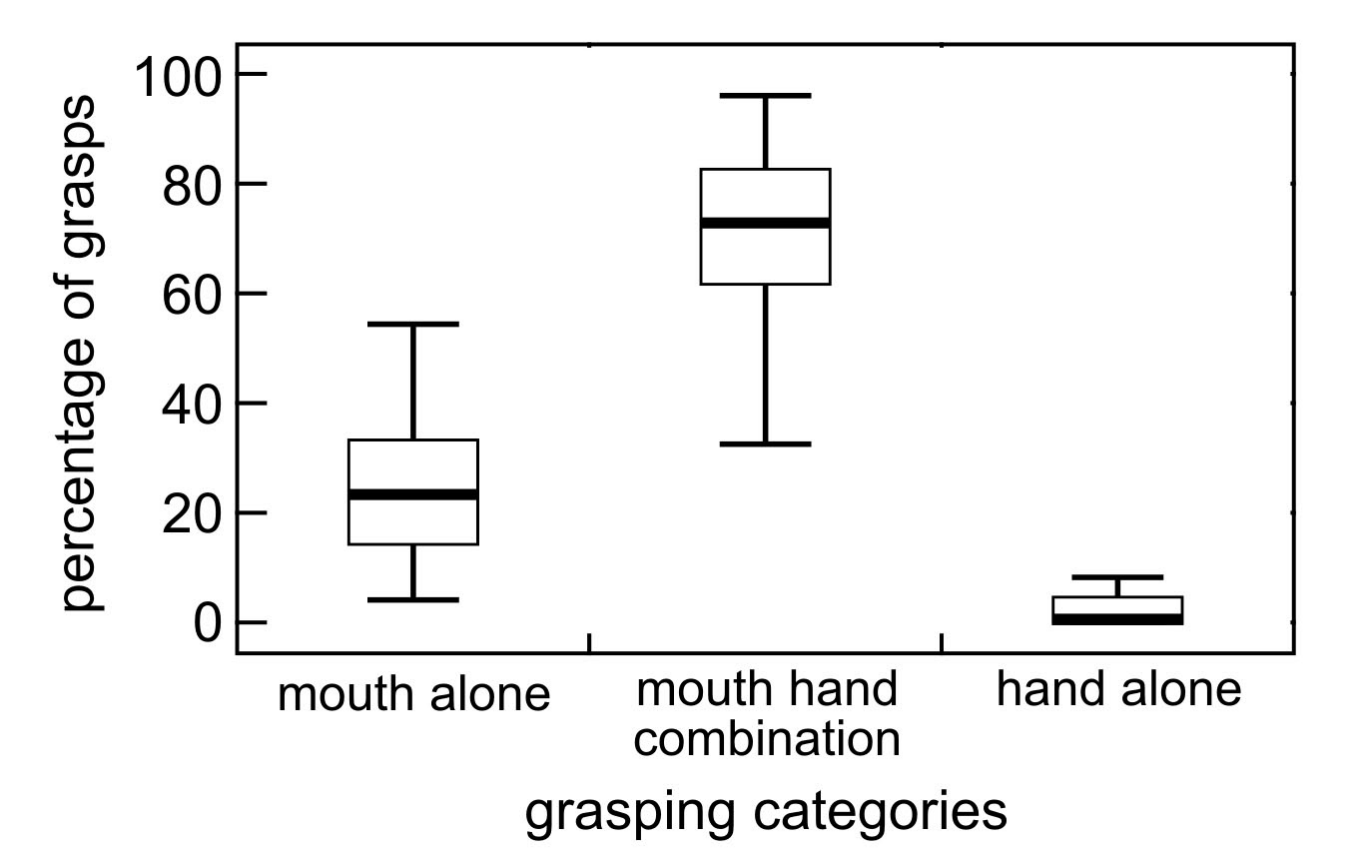
**Percentage of grasps with the mouth alone, a hand-mouth combination or with the hand alone**.

In the SGT task, subjects (N = 37) showed a significant difference in the usage of the three grasping categories: Hand-mouth combination (mean_hand-mouth _= 69.0%; SD = 19.5%), mouth alone (mean_mouth alone _= 28.1%, SD = 20.2%) and hand alone (mean_hand alone _= 2.9%, SD = 4.4%; Friedman-test: χ^2 ^= 60.33, df = 2, N = 37, p < 0.001, Figure 2). They used a hand-mouth combination significantly more often than the mouth or hand alone to grasp a mealworm (Wilcoxon-test: hand-mouth *versus *mouth alone: T = 11.25, n = 37, p < 0.001; hand-mouth *versus *hand alone: T = 0, n = 37, p < 0.001). Further, they used the mouth significantly more than the hand alone (Wilcoxon-test: mouth alone *versus *hand alone: T = 5, n = 35, p < 0.001). Due to the limited sample size of grasping acts using one hand alone, it was not possible to analyze the HI or ABS-HI for this task. There were no significant differences in the usage of the three grasping categories between sexes (Mann-Whitney-U≥116, N_m _= 15, N_f _= 22, p ≥ 0.129) and there was also no correlation between age and the three grasping categories (Spearman correlation: r_s _≤ |0.188|, N = 37, p ≥ 0.264).

### Postural tasks

In the FGT-sit task, 42 of the subjects (N = 54; 77.8%; Table 1) showed an individual hand preference by using one hand significantly more often than the other (Binominal test: p ≤ 0.05): 24 subjects were right-handed and 18 subjects were left-handed. The number of lateralized subjects was significantly higher than expected by chance (Chi-Square = 18, df = 2, N = 54, p < 0.001). However, no population level hand preference was found since the number of left- and right-handed subjects was not significantly different from chance (Binomial test: p = 0.441). Also, a one-sample t-test indicated that the mean HI_sit _score per subject (mean_sit _= 0.07, SD = 0.78) did not differ significantly from chance level (one-sample t-test: t = 0.694, df = 53, p = 0.491). There was no significant difference in the HI_sit _and ABS-HI_sit _between the sexes (Mann-Whitney-U≥323.5, N_m _= 23, N_f _= 31, p ≥ 0.557) and also no correlation between age and HI_sit _or ABS-HI_sit _(Spearman correlation: r_s _≤ |0.299|, N = 54, p ≥ 0.096).

**Table 1:** Summary of statistical data for the four postural tasks.

Tasks	Sit	Biped	Cling	Triped
Total	54 (23,31)	31 (13,18)	31 (13,18)	29 (12,17)

R	24 (11,13)	15 (8,7)	9 (4,5)	8 (2,6)

L	18 (7,11)	13 (4,9)	16 (5,11)	16 (8,8)

A	12 (5,7)	3 (1,2)	6 (4,2)	5 (2,3)

P of Chi-Square test (50%A:25%L:25%R)	** < 0.000**	** < 0.000**	**≤0.001**	** < 0.000**

P of Binomial test (50%R:50%L)	0.441	0.851	0.230	0.152

HI	0.07 ± 0.78	-0.02 ± 0.86	-0.16 ± 0.83	0.21 ± 0.74

ABS-HI	0.72 ± 0.30	0.81 ± 0.23	0.78 ± 0.29	0.71 ± 0.26

P of t-test on HI	0.491	0.920	0.297	0.130

Number of right-handed (R), left-handed (L) and ambiguous (A) subjects; in brackets: Number of males, number of females; p-value of the Chi Square and Binomial test; mean handedness indices (HI) and absolute handedness indices (ABS-HI) and the p-value for the one-sample t-test.

In the FGT-biped task, 28 of the subjects (N = 31; 90.3%; Table 1) showed an individual hand preference by using one hand significantly more often than the other (Binominal test: p ≤ 0.05): 15 subjects were right-handed and 13 subjects were left-handed. The number of lateralized subjects was significantly higher than expected by chance (Chi-Square = 20.4, df = 2, N = 31, p < 0.001). However, no population level hand preference was found since the number of left- and right-handed subjects was not different from chance (Binomial test: p = 0.851). Also, a one-sample t-test indicated that the mean HI_biped _score per subject (mean_biped _= -0.02, SD = 0.86) did not differ significantly from chance (one-sample t-test: t = -0.101, df = 30, p = 0.920). There was no significant difference in the HI_biped _and ABS-HI_biped _between the sexes (Mann-Whitney-U≥76, N_m _= 13, N_f _= 18 p = 0.093) and also no significant correlation between age and HI_biped _(Spearman correlation: r_s _= 0.141, N = 31, p = 0.449). In contrast, there was a correlation between age and ABS-HI_biped _(Spearman correlation: r_s _= -0.408, N = 31, p = 0.023).

In the FGT-cling task, 25 of the subjects (N = 31; 80.7%; Table 1) showed an individual hand preference by using one hand significantly more often than the other (Binominal test: p ≤ 0.05): 9 subjects were right-handed and 16 subjects were left-handed. The number of lateralized subjects was significantly higher than expected by chance (Chi-Square = 14.81, df = 2, N = 31, p ≤ 0.001). However, no population level hand preference was found since the number of left- and right-handed subjects was not different from chance (Binomial test: p = 0.230). Also, a one-sample t-test indicated that the mean HI_cling _score per subject (mean_cling _= -0.16, SD = 0.83) did not differ significantly from chance (one-sample t-test: t = -1.062, df = 30, p = 0.297). There was no significant difference in the HI_cling _and ABS-HI_cling _between the sexes (Mann-Whitney-U≥79, N_m _= 13, N_f _= 18, p ≥ 0.110) and also no correlation between age and HI_cling _and ABS-HI_cling _(Spearman correlation: r_s _≤ |0.171|, N = 31, p ≥ 0.357).

In the FGT-triped task, 24 of the subjects (N = 29; 82.8%; Table 1) showed an individual hand preference by using one hand significantly more often than the other (Binominal test: p ≤ 0.05): 8 subjects were right-handed and 16 subjects were left-handed. The number of lateralized subjects was significantly higher than expected by chance (Chi-Square = 16.9, df = 2, N = 29, p < 0.001). However, no population level hand preference was found since the number of left- and right-handed subjects was not different from chance (Binomial test: p = 0.152). Also, a one-sample t-test indicated that the mean HI_triped _score per subject (mean_triped _= -0.21, SD = 0.74) did not differ significantly from chance (t = -1.559, df = 28, p = 0.130). There was no significant difference in the HI_triped _and ABS-HI_triped _between the sexes (Mann-Whitney-U≥72, N_m _= 12, N_f _= 17, p ≥ 0.183) and also no correlation between age and HI_triped _and ABS-HI_triped _(Spearman correlation: r_s _≤ |0.234|, N = 29, p ≥ 0.220).

### Comparison of postural tasks

**Figure 3:**
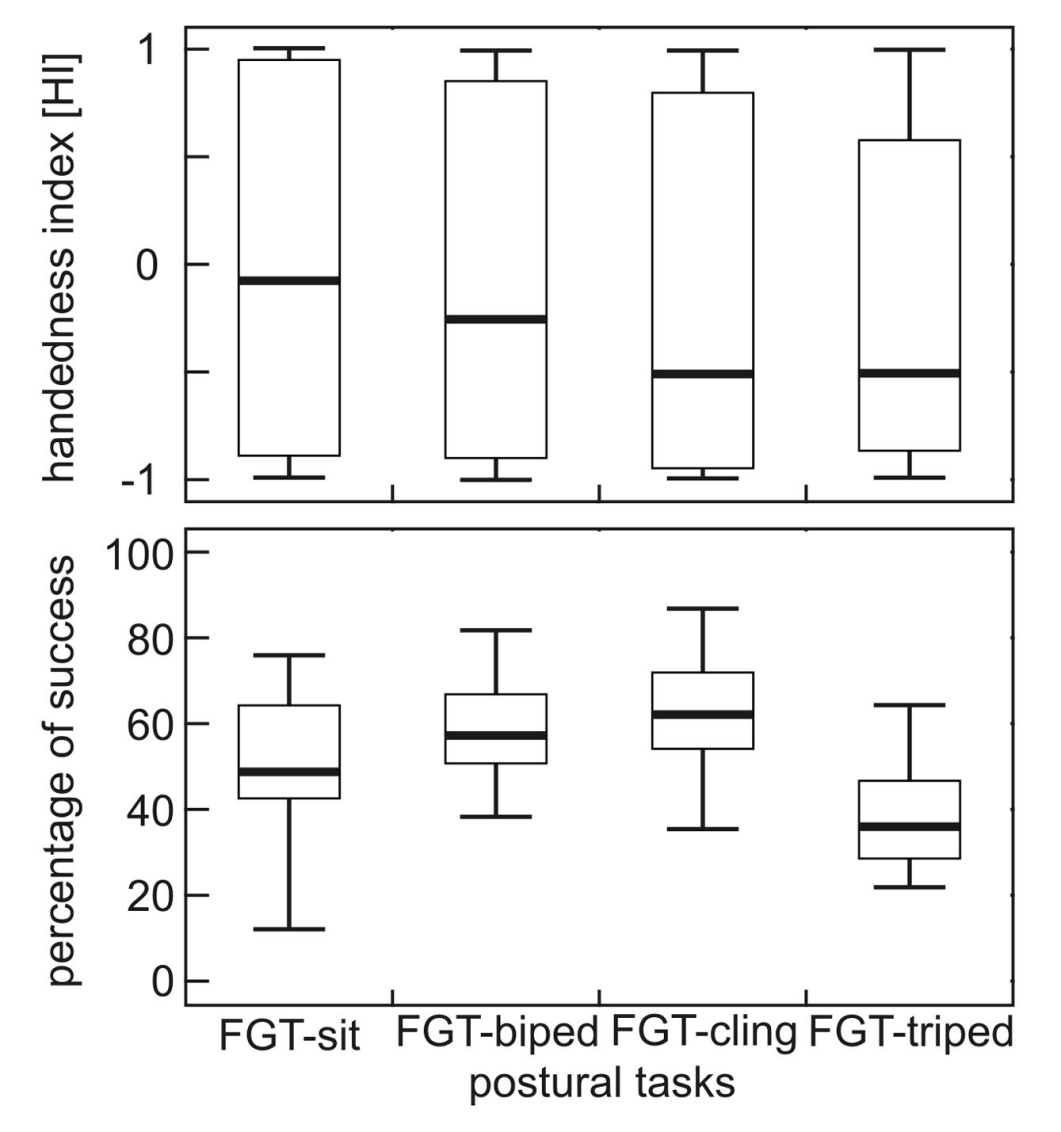
**Mean handedness index (A) and success rate (B) for the four postural tasks; based on equal sample size (N = 27)**.

We compared the HI and ABS-HI between the four postural tasks for the 27 subjects that participateding in all four tasks, but found no significant differences (Friedman-test: χ^2^≤5.6, df = 3, N = 27, p ≥ 0.133, Figure 3a). Also, the number of lateralized *versus *non-lateralized subjects did not differ significantly between the four postural tasks (Cochran's Q = 2.0, df = 3. N = 27, p = 0.572) suggesting that posture did not influence the direction and strength of hand preference.

Comparing the direction of hand preference 17 of 27 subjects showed a consistent hand preference for all four postural tasks (10 left-handed; 7 right-handed; see Additional file 6: Table HI). Only two subjects switched the direction of hand preference from one task to another task. Eight subjects showed a consistent hand preference for at least two tasks and were ambiguous for the remaining tasks (see Additional file 6: Table HI).

The HIs of the four postural tasks showed a significant strong positive correlation with one another (Spearman correlation: r_s _≥ 0.786, N = 27, p < 0.001). The analysis of the ABS-HI indicated significant positive correlations between biped and cling (Spearman correlation: r_s _= 0.678, N = 27, p < 0.001).

### Level of difficulty of the postural tasks

To measure the level of difficulty of the postural tasks we used the percentage of successful hand grasps (= success rate). The success rate differed significantly between tasks (Friedman-test: χ^2 ^= 45.15, df = 3, N = 27, p < 0.001, Figure 3b). Pair wise comparisons showed that the FGT-triped was significantly more difficult for the subjects than the other three postural tasks (Wilcoxon-test: T ≤8.8, n = 27, p < 0.001 for all comparisons). Further, the FGT-sit task was significantly more difficult than the FGT-biped and FGT-cling task (Wilcoxon-test: sit *versus *biped: T = 11.86, n = 26, p = 0.019; sit *versus *cling T = 8.4, n = 27, p < 0.001), whereas there was no significant difference between the FGT-biped and FGT-cling task (Wilcoxon-test: T = 13.22, n = 26, p = 0.151).

## Discussion

We found that in the simple food grasping task (SGT) mouse lemurs prefer to use combinations of mouth and hand or the mouth alone to pick up mealworms over using one hand alone. Nevertheless, if the use of the mouth was prevented, mouse lemurs showed individual hand preference, but no population level hand preference in all four postural tasks. We found no significant differences in the direction and strength of hand preference between the four postural tasks. The majority of subjects showed consistent hand preference in all postural tasks. Further, we found significant positive correlations for the direction of hand preference between the postural tasks. Although hand preference did not differ between the postural tasks, we found differences in their level of difficulty, suggesting the following order: triped > sit > cling = biped.

In the simple food grasping task, reflecting the natural foraging environment, mouse lemurs prefer to use the mouth in combination with the hands. Mouse lemurs catch the mealworms with rapid strikes by stretching out both hands to hold the mealworm and to pick it up with the mouth. This finding agrees with previous findings in gray mouse lemurs, based on a smaller sample size [32,33]. The preferred use of the mouth was also shown in other primate species such as the dwarf lemurs [32,33], greater galagos [32,33], marmosets [22] and sifakas [40], whereas lesser galagos [32,33] and apes [41] preferred to use a single hand to reach for food. There are two potential explanations. First, differences in grasping abilities based on anatomical differences could be related to the usage of the mouth. Lemelin & Jungers [42] found an inverse relationship between hand morphology, reflecting different degrees of prehensility, and body size. As body size increases there is a decrease in phalangeal indices which probably results in different grasping abilities. *Microcebus murinus *is characterized by hands with longer fingers relative to the palm compared to larger, more frugivorous prosimians. Further, while catching insects, small-bodied mouse lemurs have to catch moving insects that are too large to handle with only one hand. Therefore, the combined use of mouth and hand makes them more successful in foraging. This is also supported by the high success rate of 98% in the SGT task compared to a low success rate ranging from 37.5 to 60.9% in the FGT task where mouth usage was prevented. Rogers [36] suggested that the whole-hand snatch-grasping by prosimians did not differ from the usage of paws by non-primate mammals. She suggested that internal control for fine motoric function only evolved in some primates. Further, Hopkins and colleagues [43,44] argued that the use of different grasping techniques due to anatomical differences between species, is an important factor in determining hand preference. Second, the preferred usage of the mouth could be affected by different feeding strategies. Mouse lemurs feed not only on fruits and insects but also on gum [45-47]. Gum feeding is especially prominent in the dry season. Mouse lemurs use their teeth to scratch tree bark and lick the gum, a process which does not require hand usage [46]. This is in agreement with findings of Singer & Schwibbe [22]. They observed that *Callithrix*, which feed on exudates, showed a strong preference for the mouth to pick up food items. In contrast, lion tamarins, which are specialized in using manipulation and extracting insects showed a preference for hand usage in the same study.

Our findings of manual lateralization at an individual, but not at a population level in mouse lemurs are in agreement with previous findings for the FGT-sit [10,34,35] and biped task [10] and are new for the triped and cling task. Further, we did not find any influence of body posture on the direction or strength of hand preference in gray mouse lemurs, supporting previous findings in mouse lemurs for the comparison of the sit versus biped task, based on a small sample size [10]. The *postural origin theory *[6] was not supported by our results. This hypothesis is based on the assumption, that one hand (the right- hand) is needed for postural support, whereas the other hand (the left hand) is used for reaching. However, in the FGT-cling task, mouse lemurs were also able to support their posture only with their feet, whereas both hands were free and equally available for food reaching. This could explain why mouse lemurs do not establish a population bias or an increase in the strength of hand preference for the FGT-cling task. In addition, our results are not in agreement with the *bipedalism theory *which proposed an increase in the strength of hand preference from a stable (FGT-sit, FGT-triped) to an unstable posture (FGT-biped) [8,9]. We have to mention that in the bipedal task subjects were not free standing but showed postural support with one hand while the other was picking up the food item. Therefore, it could be argued that in a free-standing bipedal posture the strength of hand preference could be increased. Further, the results of the bipedal task could be affected by the proportion of activities subjects spent in a bipedal posture. Since mouse lemurs naturally spend less time in bipedal postures it could be argued that manual laterality is less influenced by this posture. However, the lack of postural influence on hand preference was also observed in Callitrichinae [22]. It could be assumed that in species where posture has an influence on hand preferenc this relies on different levels of difficulty induced by these postures [36].

Although the grasping behaviour itself was similar across all unimanual tasks, i.e. simple reaching for a food item during all postural tasks, we found significant differences in the success rates between the tasks, indicating that body postures differ in their level of difficulty. However, the level of difficulty did not affect the direction and strength of hand preferences. It was surprising that the triped task, which was similar to the quadrupedal task in other studies, was the most difficult task for the subjects. However, this could be explained by methodological reasons rather than by body posture alone. Since subjects did not use the hand to pick up mealworms in a simple food grasping task that would be equivalent to the quadrupedal task in other species, we were forced to develop an apparatus which forced the subjects to use their hands. In the FGT-sit, FGT-biped and FGT-cling task the box was placed in front of the subjects, forcing them to use a horizontal movement to pick up the mealworm. In the FGT-triped task the box was placed below the subjects, forcing them to use a vertical movement. Since it is assumed that mouse lemurs lack fine motoric control of their hands, the different movement axis could result in different success rates. The result that the sit task was more difficult than the biped or cling task could be explained by the fact that this was the first task animals were confronted with. However, Leliveld et al. [35] showed that task experience did not influence the HI or ABS-HI in gray mouse lemurs. Interestingly, they showed a 98% success in the simple food grasping task which also stressed the advantage of using the mouth-hand combination and the lower importance of the hand.

We found no influence of sex on the direction and strength of hand preference. Further, we can not support the theory that the usage of the mouth decreases with age or that the strength of hand preference increases with age. For the FGT-biped task the strength of manual laterality decreased with age. A speculative hypothesis could be that younger individuals show more temperament (i.e. more hasty and less concentrated) than older subjects which could result in a stronger degree of laterality [35,36,48,49].

Further, it could be argued that using other measurements favors different results [50-52]. Therefore, we recalculated our results using other often published measurements such as the Z-score or hand performance. However, we obtained similar results using the Z-score or the Binomial test in the decision whether a subject was ambiguous, right- or left-handed (only one subject changed from ambiguous to right-handed for the sit task). Hand preference (i.e. the hand spontaneously chosen for a specific task) used in this study is the most commonly used measure for manual lateralization, but several authors suggested that successful hand preference (i.e. the hand which is more successful in completing a specific task) gives a better indication of motor lateralization (e.g., [50,51]) and is less affected by repetitive use [51]. Therefore, we also calculated successful hand preference and total hand preference (i.e. total number of grasping events). However, we obtained similar results and the three measurements showed strong correlations (Spearman correlation: sit: r_s _≥ 0.916, N = 54, p < 0.001; biped: r_s _≥ 0.922, N = 31, p < 0.001; cling: r_s _≥ 0.881, N = 31, p < 0.001; triped: r_s _≥ 0.886, N = 29, p < 0.001).

All in all, mouse lemurs prefer mouth-hand combinations or the mouth to retrieve food in a natural foraging situation. In contrast to other prosimians, they show a lesser degree of manual laterality since no population level handedness was observed in any of the four postural tasks. This supports the hypothesis that the role of the mouth is a critical factor for the development of manual lateral bias [53]. Ward et al. [32] showed that there is a negative correlation between the percentage of mouth use and the strength of lateral bias which means that when primate species use their mouths more, they show fewer hand preferences. Olson et al. [14] found that gibbons and gorillas which moved more often bipedal than orang-utans showed stronger hand preferences. They proposed that the degree of bipedality a species exhibits in the natural environment is related to the strength of hand preference and to the occurrence of population level handedness. Therefore, it can be assumed that ecological adaptation indicated by postural habit has an important impact on the development of manual laterality.

## Conclusion

To conclude, this study shows that in a natural foraging situation gray mouse lemurs prefer to use their mouths or a hand-mouth combination. Nevertheless, in a foraging task where mouth usage was prevented they show individual hand preferences, but no population level hand preference independent of task-specific body posture. Our results support the hypothesis that small-bodied, quadrupedal primates with a horizontal orientation to the trunk prefer mouth retrieval of food and are less manually lateralized than large-bodied species which consume food in a more upright, and less stable, body posture. Therefore, we hypothesize that ecological adaptation indicated by the postural habit and body size shaped the evolution of manual laterality.

## Authors' contributions

MS participated in the design of the study, conducted the experiments, performed the video and statistical analyses and prepared the manuscript. MJ contributed to designing the study and the preparation of the manuscript. LL conducted part of the experiment, performed part of the video analysis and contributed to the preparation of the manuscript. EZ initiated, financed, mentored the study and contributed to designing the study and the preparation of the manuscript. All authors have read and approved this manuscript.

## Supplementary Material

Additional file 1 **Movie SGT**. Example of an experimental trial of the simple food grasping task (SGT). Click here for file

Additional file 2 **Movie FGT-sit**. Example of an experimental trial of the FGT-sit task. Click here for file

Additional file 3 **Movie FGT-biped**. Example of an experimental trial of the FGT-biped task. Click here for file

Additional file 4 **Movie FGT-cling**. Example of an experimental trial of the FGT-cling task. Click here for file

Additional file 5 **Movie FGT-triped**. Example of an experimental trial of the FGT-triped task. Click here for file

Additional file 6 **Table HI**. Handedness index (HI) and handedness bias (bias) for each subject and for each postural task; R - right-handed; L - left-handed; A - ambiguous; m - males, f-females; bold subjects showed consistent hand preference for all four postural tasks; data for the FGT-sit task were already published in ^1 ^[34], ^2 ^[35], from this data the first three sessions were selected to keep the number of sessions constant through the study. Click here for file
