# Supplementary material for: Does body posture influence hand preference in an ancestral primate model?
Source: BMC Evol Biol. 2011 Feb 28;11:52. doi: 10.1186/1471-2148-11-52 (PMC3056780; doi:10.1186/1471-2148-11-52)
Supplement: Additional file 6 — Table HI. Handedness index (HI) and handedness bias (bias) for each subject and for each postural task; R - right-handed; L - left-handed; A - ambiguous; m - males, f-females; bold subjects showed consistent hand preference for all four postural tasks; data for the FGT-sit task were already published in 1 [34], 2 [35], from this data the first three sessions were selected to keep the number of sessions constant through the study. [file 1471-2148-11-52-S6.DOC]

## Additional file 6 – Table HI: Handedness index (HI) and handedness bias (bias) for each subject and for each postural task; R – right-handed; L – left-handed; A – ambiguous; m – males, f-females; bold subjects showed consistent hand preference for all four postural tasks; data for the FGT-sit task were already published in ^1^ [34], ^2^ [35]; from this data the first three sessions were selected to keep the number of sessions constant through the study.

| TASK |  | SIT | | BIPED | | CLING | | TRIPED | |
| --- | --- | --- | --- | --- | --- | --- | --- | --- | --- |
|  | sex | HI | bias | HI | bias | HI | bias | HI | bias |
| **Strolch** | **m** | **1.00** | **R** | **0.89** | **R** | **1.00** | **R** | **0.92** | **R** |
| **Tiger**^2^ | **m** | **-1.00** | **L** | **-0.90** | **L** | **-0.90** | **L** | **-1.00** | **L** |
| Uli^1,2^ | m | -1.00 | L | -0.26 | A | -0.65 | L | -0.91 | L |
| Urban^1,2^ | m | 0.16 | A | 0.60 | R | -0.45 | A | -0.52 | L |
| **Uriel**^1,2^ | **m** | **-0.79** | **L** | **-1.00** | **L** | **-1.00** | **L** | **-1.00** | **L** |
| Urs^2^ | m | 1.00 | R | 0.78 | R | 0.90 | R | -0.24 | A |
| Vincent^1,2^ | m | 0.55 | R | 0.76 | R | 0.43 | A | 0.25 | A |
| **Virgil**^1,2^ | **m** | **-1.00** | **L** | **-1.00** | **L** | **-0.91** | **L** | **-1.00** | **L** |
| Vito | m | -0.33 | A | 0.76 | R | 0.30 | A | -0.48 | L |
| **Yves**^1,2^ | **m** | **0.91** | **R** | **1.00** | **R** | **0.92** | **R** | **0.48** | **R** |
| **Zambo**^1,2^ | **m** | **-0.73** | **L** | **-0.73** | **L** | **-0.89** | **L** | **-1.00** | **L** |
| **Rosa** | **f** | **-1.00** | **L** | **-1.00** | **L** | **-1.00** | **L** | **-0.70** | **L** |
| **Saphira** | **f** | **-0.82** | **L** | **-1.00** | **L** | **-1.00** | **L** | **-0.60** | **L** |
| Sina | f | 1.00 | R | 1.00 | R | 0.20 | A | 0.25 | A |
| Susi | f | -0.30 | A | -1.00 | L | -1.00 | L | -0.40 | A |
| **Tanja** | **f** | **-0.90** | **L** | **-0.92** | **L** | **-1.00** | **L** | **-0.52** | **L** |
| Tasha^2^ | f | 0.74 | R | 0.60 | R | 0.24 | A | 0.67 | R |
| **Tipi** | **f** | **-1.00** | **L** | **-0.89** | **L** | **-0.83** | **L** | **-0.83** | **L** |
| **Tweety**^2^ | **f** | **0.75** | **R** | **0.81** | **R** | **0.70** | **R** | **0.71** | **R** |
| **Uma**^1,2^ | **f** | **1.00** | **R** | **1.00** | **R** | **1.00** | **R** | **1.00** | **R** |
| **Undine**^1,2^ | **f** | **-0.89** | **L** | **-0.83** | **L** | **-0.91** | **L** | **-0.58** | **L** |
| Urania^1,2^ | f | 0.38 | A | -1.00 | L | -1.00 | L | -1.00 | L |
| **Ursel** | **f** | **1.00** | **R** | **1.00** | **R** | **1.00** | **R** | **1.00** | **R** |
| **Ursina**^1,2^ | **f** | **1.00** | **R** | **1.00** | **R** | **1.00** | **R** | **1.00** | **R** |
| **Vicky**^1,2^ | **f** | **1.00** | **R** | **0.92** | **R** | **1.00** | **R** | **0.90** | **R** |
| **Vivian**^1,2^ | **f** | **-0.91** | **L** | **-0.85** | **L** | **-1.00** | **L** | **-0.68** | **L** |
| Wilma^1,2^ | f | -0.08 | A | -0.30 | A | -0.52 | L | -0.92 | L |
| Adam^1,2^ | m | 0.30 | A |  |  |  |  |  |  |
| Adrian^1,2^ | m | 0.57 | R |  |  |  |  |  |  |
| Amigo^1,2^ | m | 0.80 | R |  |  |  |  |  |  |
| Beetle^1,2^ | m | 0.67 | R |  |  |  |  |  |  |
| Bibo^1,2^ | m | 0.53 | R |  |  |  |  |  |  |
| Daniel^1,2^ | m | 0.80 | R |  |  |  |  |  |  |
| Emil^1,2^ | m | -1.00 | L |  |  |  |  |  |  |
| Rocky | m |  |  |  |  |  |  | -0.53 | L |
| Wotka^1,2^ | m | 0.78 | R |  |  |  |  |  |  |
| Wutz^1,2^ | m | 1.00 | R |  |  |  |  |  |  |
| Xaver^1,2^ | m | 0.00 | A | 0.76 | R | 1.00 | R |  |  |
| Yeti^1,2^ | m | -0.18 | A | 0.44 | R | -0.04 | A |  |  |
| Zafy^1,2^ | m | -0.88 | L |  |  |  |  |  |  |
| Anna^1,2^ | f | -0.58 | L |  |  |  |  |  |  |
| Asta^1,2^ | f | -1.00 | L |  |  |  |  |  |  |
| Athena^1,2^ | f | 1.00 | R |  |  |  |  |  |  |
| BB^1,2^ | f | 0.73 | R |  |  |  |  |  |  |
| Bijou^1,2^ | f | 0.00 | A |  |  |  |  |  |  |
| Bonnie^2^ | f | 0.73 | R |  |  |  |  |  |  |
| Romy | f |  |  |  |  |  |  | -0.44 | A |
| Vangi^1,2^ | f | -1.00 | L |  |  |  |  |  |  |
| Wolke^2^ | f | -0.89 | L |  |  |  |  |  |  |
| Xara^1,2^ | f | 0.79 | R |  |  |  |  |  |  |
| Zita^1,2^ | f | 0.36 | A |  |  |  |  |  |  |
| Zizi^1,2^ | f | 0.83 | R |  |  |  |  |  |  |
| Zoly | f | 0.65 | R | -0.20 | A | -0.52 | L |  |  |
| Zottie^1,2^ | f | 0.47 | A |  |  |  |  |  |  |
| Zuby^1,2^ | f | -0.89 | L | -0.92 | L | -1.00 | L |  |  |
| Zwipsy^2^ | f | -0.33 | A |  |  |  |  |  |  |
